# A Novel Fuzzy Multilayer Perceptron (F-MLP) for the Detection of Irregularity in Skin Lesion Border Using Dermoscopic Images

**DOI:** 10.3389/fmed.2020.00297

**Published:** 2020-07-07

**Authors:** Abder-Rahman Ali, Jingpeng Li, Summrina Kanwal, Guang Yang, Amir Hussain, Sally Jane O'Shea

**Affiliations:** ^1^Faculty of Natural Sciences, Computing Science and Mathematics, University of Stirling, Stirling, United Kingdom; ^2^Department of Computing and Informatics, Saudi Electronic University, Al-Dammam, Saudi Arabia; ^3^National Heart and Lung Institute, Imperial College London, London, United Kingdom; ^4^Cognitive Big Data and Cybersecurity Research Lab, Edinburgh Napier University, Edinburgh, United Kingdom; ^5^Mater Private Hospital, Cork, Ireland

**Keywords:** melanoma, irregularity, dermoscopy, multilayer perceptron, fuzzy logic

## Abstract

Skin lesion border irregularity, which represents the B feature in the ABCD rule, is considered one of the most significant factors in melanoma diagnosis. Since signs that clinicians rely on in melanoma diagnosis involve subjective judgment including visual signs such as border irregularity, this deems it necessary to develop an objective approach to finding border irregularity. Increased research in neural networks has been carried out in recent years mainly driven by the advances of deep learning. Artificial neural networks (ANNs) or multilayer perceptrons have been shown to perform well in supervised learning tasks. However, such networks usually don't incorporate information pertaining the ambiguity of the inputs when training the network, which in turn could affect how the weights are being updated in the learning process and eventually degrading the performance of the network when applied on test data. In this paper, we propose a fuzzy multilayer perceptron (F-MLP) that takes the ambiguity of the inputs into consideration and subsequently reduces the effects of ambiguous inputs on the learning process. A new optimization function, the fuzzy gradient descent, has been proposed to reflect those changes. Moreover, a type-II fuzzy sigmoid activation function has also been proposed which enables finding the range of performance the fuzzy neural network is able to attain. The fuzzy neural network was used to predict the skin lesion border irregularity, where the lesion was firstly segmented from the skin, the lesion border extracted, border irregularity measured using a proposed measure vector, and using the extracted border irregularity measures to train the neural network. The proposed approach outperformed most of the state-of-the-art classification methods in general and its standard neural network counterpart in particular. However, the proposed fuzzy neural network was more time-consuming when training the network.

## 1. Introduction

The increasing incidence of melanoma renders the attempts of the early detection of melanoma a continuing public health priority. Despite its aggressive infiltration of other body parts, melanoma is highly curable if diagnosed early and treated timely (1). Early detection is crucial since it contributes to a better survival; the 5-year survival rate for early stage invasive melanoma is 94%, compared to a 5-year survival rate of only 17% for melanomas that have spread to other parts of the body. There is a niche to develop an objective, bedside tool that could be used as an adjunct in the clinical assessment of skin lesions. Tracking tumor changes manually is also labor-intensive, especially for patients with multiple moles on their skin.

The ABCD rule (2) emerged in 1985 by a group of researchers at the New York University as a simple framework that physicians, novice dermatologists, and non-physicians could use to learn about the features of melanoma in its early curable stage, enhancing thereby the early detection of melanoma. The rule is more geared toward the public than the 7-point checklist which was designed for non-dermatological *medical* personnel. The approach has then been verified by the 1992 National Institutes of Health Consensus Conference Report on Early Melanoma, in addition to other studies published at the time (3–6), and is being advertised by the American Cancer Society as a method to help in seeking early medical evaluation of any suspicious pigmented lesions. The ABCD acronym refers to four parameters: (i) Asymmetry, (ii) Border irregularity, (iii) Color variegation, (iv) Diameter >6 mm. Such parameters provide simple means for appraisal of pigmented cutaneous lesions that may need to be further examined by a specialist, which might result on further work of dermoscopy or biopsy, or both. The rule is basically designed to be used on a daily life basis by both the layperson and the primary care physician (non-dermatologist) as a simple method to alert on the clinical features of melanoma, and is intended to help explain a subset of melanomas called *thin tumors* which could otherwise be confused with benign pigmented lesions.

One of the most significant factors in melanoma diagnosis is *border irregularity* (the B feature in the ABCD rule) (7). As opposed to benign pigmented lesions which tend to possess regular borders, melanoma lesions have irregular borders due to the uneven growth rate (8), the spread of melanocytes in various directions, and the regression of invasion and/or genetic instability of the lesion (9). In this paper we proposes a type-II fuzzy logic based multilayer perceptron that considers the ambiguity of neurons and attempts to reduce the effects of such ambiguous data on the network learning process. Such network will be used in detecting the skin lesion border irregularity and will be compared with its standard neural network counterpart. Detecting the other features (i.e., ACD) is explained in our other work (10).

Section 2 reviews related work, section 3 introduces the concepts of the perceptron, multilayer perceptron, and gradient descent, section 4 explains the notions of fuzzy sets and type-II fuzzy sets, fuzzy c-means clustering is explained in section 5, the proposed fuzzy multilayer perceptron (F-MLP) is described in section 6, sections 7–9 describe the skin lesion segmentation process, how we detect the skin lesion border, and how the border irregularity is measured, respectively, results are depicted in section 10, and the paper is concluded in section 11.

## 2. Related Work

Although Artificial Neural Networks (ANNs) have been proved to work well with supervised learning tasks, they do not include information related to the ambiguity of the inputs. This issue can have a negative effect on how the weights are being updated in the learning process, and subsequently affecting the accuracy of the network results. The term *fuzzy neural network* was proposed in 1975 (11) when the authors attempted to extend the McCulloch-Pitts model of the neuron (12) in such a way that allows the activity of a neuron to be fuzzy rather than an all-or-none process. Different studies have then been published on combining fuzzy logic and neural networks. Keller and Hunt (13) proposed a fuzzy perceptron (the building block of fuzzy neural networks) to alleviate the major drawback with the crisp perceptron which is its inability to terminate when the data is not linearly separable. Goh et al. (14) developed an enhanced fuzzy perceptron that demonstrates higher stability and functionality compared to the fuzzy perceptron. A neural network classifier which uses the min-max hyperboxes as fuzzy sets aggregated into fuzzy set classes was introduced in Simpson (15), and was referred to as a fuzzy min-max classification neural network. As opposed to this supervised learning approach, an unsupervised learning pattern clustering sibling to this work, namely fuzzy min-max clustering neural network was proposed in Simpson (16). A fuzzy neural network based on the multilayer perceptron and capable of fuzzy classification of patterns has been proposed in Pal and Mitra (17) and Mitra et al. (18). Fuzzified neural networks, where fuzzy numbers are used for inputs, outputs, and/or connection weights have been proposed in Buckley and Hayashi (19), Ishibuchi et al. (20), Ishibuchi (21). Researchers attempted to enhance the fuzzy perceptron; Chen and Chang (22) proposed a fuzzy perceptron that addresses classification problems where it is capable of accepting two different kinds of input data: numerical data and fuzzy IF-THEN rules. Chen and Chen (23) proposed a fuzzy kernel perceptron where the fuzzy perceptron and the Mercer Kernels (24) are incorporated, such that input data is first mapped into a high-dimensional feature space and the fuzzy perceptron is then utilized in order to find a linear separating hyperplane in the high-dimensional feature space. A comprehensive review of proposed neurofuzzy systems in the periods 2002–2012 can be found in Samarjit et al. (25). Lixin Fan from Nokia Technologies wrote a detailed guide (26) that aims to bridge the gap between fuzzy logic and deep learning (64).

In this paper we propose a fuzzy multilayer perceptron (F-MLP) that uses a developed fuzzy gradient descent which incorporates the membership degrees of neurons (obtained using fuzzy c-means clustering) to reduce the effects of ambiguous neurons on the neural network learning process. Moreover, a proposed type-II fuzzy sigmoid activation function is used which allows to represent the range (lowest and highest) of performance the fuzzy neural network is able to achieve.

A radial search algorithm (27) was used to detect the skin lesion border in Golston et al. (28), where different sliding windows that represent the origin of a radii are automatically detected in the skin lesion. Sufficiently high jumps in luminance (also contain sufficiently sustained luminance) are searched for in the radii to form the candidate border points (29). Irregularity was eventually found using the *irregularity index*: $I=\frac{P^{2}}{4\pi A}$, where *P* and *A* are the perimeter (number of points on the detected border) and area (number of points on and within the border) of the closed boundary, respectively. Borders with an irregularity index greater than 1.8 were classified as being irregular. Sixty skin tumor images were labeled by a dermatologist as being regular or irregular (regular: 14, irregular: 46). 83.3% of the tumors were classified correctly (8/14 and 42/46 for regular and irregular borders, respectively).

Ng and Lee (30) used fractal dimensions (FDs) in measuring the irregularity of skin lesion borders. Four fractal dimension measures were found for each color image: direct FD, vertical smoothing FD, horizontal smoothing FD, and multi-fractal dimension of order two. Those FDs were also calculated on the blue band of the images. Four hundred and sixty eight melanocytic lesions (not hairy) have been segmented using a multi-stage method (31) and used to test the proposed approach. Results showed that the multi-fractal method performed the best. FDs were also used in Claridge et al. (32) and Ali et al. (33).

An approach which analyzes the structural irregularity of cutaneous melanocytic lesions was proposed in Lee et al. (34). The algorithm consists of two stages: (i) pre-processing: dark thick hair is removed by DullRazor (35) and the lesion border is extracted from the skin image, (ii) sigma-ratio: this is a measure derived from the scale-space filtering technique and used to analyze the structural shape of the lesion border. Results revealed that sigma-ratio is sensitive to structural indentations and protrusions (i.e., provides accurate estimation for the structure irregularity) as opposed to shape descriptors such as compactness index and fractal dimension which are more sensitive to texture irregularities than structure irregularities (36). The authors also proposed a new border irregularity measure in Lee and Atkins (36), Lee et al. (37), and Lee and Claridge (9), where all indentations and protrusions are firstly located along the lesion border and a new irregularity index is measured for each indentation and protrusion. Summing up all the individual indices provides an estimation on the overall border irregularity.

A new measure of border irregularity based on *conditional entropy* was proposed by Arbisala and Claridge (38), where it was observed that the entropy increases with the degree of irregularity. The results of the proposed measure were compared with the Indentation Irregularity Index (9) on 98 skin lesions (16 were melanoma) and showed to have a better discriminatory power; ROC curve 0.76 compared to 0.73 for the Indentation Irregularity Index.

Ma et al. (39) used *wavelet decomposition* to extract the skin lesion border structure to determine whether the lesion is naevus or melanoma. The discrete wavelet transform (DWT) was used to filter the 1D border into sub-bands down to level 9, where levels 6–9 (significant levels) have shown to contain information more relevant for classifying between melanoma and benign samples. Some statistical and geometrical feature descriptors of border irregularity were also extracted at each individual sub-band. Twenty-five measurements were formed by applying six features in four significant sub-bands and one feature in a single sub-band. A combination of features was eventually fed to a back-projection neural network. Using a small training set of 9 melanomas and 9 naevi, the best classifier was obtained when the best 13 features were used.

Jaworek-Korjakowska and Tadeusiewicz (40) used a simple method to measure border irregularity, in which a semi-quantitative evaluation method was used to divide the lesion into eight similar parts where the sharp abrupt cut-off in each part has a score of 1. A maximum score of 8 is obtained if the whole border is irregular, and a score 0 is obtained if the naevus is round with no ragged borders. Melanomas tend to have scores 4–8 (41). The approach was tested on 120 skin lesion cases with border irregularity <3 and 180 skin lesion cases with border irregularity >4, achieving a 79% accuracy.

Ali et al. (33) proposed a border irregularity measure that combines fractal dimension, zernike moments, and convexity, which are represented in a 27-value vector (zernike moments produced 25 values). Fractal dimension was found using the extracted border, and zernike moments and convexity were found using the segmented image. The extracted measures were then trained on a CNN (convolutional neural network) and Gaussian naive Bayes ensemble, which is then used for the automatic detection (i.e., classification) of skin lesion border irregularity on new images. The approach achieved outstanding results, obtaining an accuracy, sensitivity, specificity, and F-score of 93.6, 100, 92.5, and 96.1%, respectively. In this paper we use a similar skin lesion border irregularity measure, but use only fractal dimension and convexity.

## 3. Perceptrons

The perceptron is normally used in supervised linear classification tasks in which a hyperplane would be tuned to fit a training dataset. This tuned hyperplane can then be used to classify new unknown samples. This is achieved by minimizing the hyperplane's error as it is applied on the training dataset through minimizing the *error function*: $\epsilon \left(w\right)=-\sum_{i\in M}t_{i}{\text{w}}^{T}{\text{x}}_{i}$, where *M* is the set of misclassified samples, and *t*_*i*_ ∈ {−1, 1} is the class of sample **x**_*i*_. If ϵ(**w**) = 0, this means that the hyperplane completely separates the classes. This minimization process is usually carried out in iterations such that after each iteration we move toward the minimum of ϵ(**w**). The **w** vector of iteration *k* + 1 is obtained as the following weight updating step: **w**_*k* + 1_ = **w**_*k*_ + Δ**w** (weight update). Equation (1) shows the learning rule used in calculating the value for updating the weights at each increment:

(1) $$\begin{array}{l} \Delta {\text{w}}_{i}=\eta \left({true}_{j}-{pred}_{j}\right)x_{i}^{j} \end{array}$$

where η is the learning rate, *true*_*j*_ is the true class label and *pred*_*j*_ is the predicted class label.

The perceptron's learning process starts by initializing the weights to small random numbers [or 0]. For each training input sample the output value is calculated and the weights are updated until a minimum error is reached (i.e., backpropagation). The main drawback of perceptrons is that they are only able to converge when the two classes can be separated by a linear hyperplane.

A *multilayer perceptron* (also called Artificial Neural Network–ANN) is composed of neurons from the input layer, one or more hidden layers of neurons, and the output layer of neurons, where the input propagates through the network layer-by-layer in the forward direction where each layer of the network contains connections to the next layer. Such network is called a *feedforward* neural network and is typically used in supervised learning. The structure of the multilayer perceptron enables it to learn complex tasks by extracting more meaningful features from the input patterns. *Gradient descent* can be used to optimize model prediction by finding the local minimum of a function (i.e., minimize the network error), and is defined as follows:

(2) $$\begin{array}{l} \text{w}=\text{w}-\eta \times \frac{d}{d\text{w}}F\left(\text{w}\right) \end{array}$$

where **w** are the weight values, η is the learning rate, and $\frac{d}{dw}F\left(\text{w}\right)$ is the derivative of the objective function *F*(**w**) representing the slope (gradient).

## 4. Fuzzy Sets

Let *U* = {*x*_1_, *x*_2_, *x*_3_, …, *x*_*n*_} be the universe of discourse, a fuzzy set *A* ∈ *U* is defined as the set of ordered pairs {(*x*_*i*_, μ_*A*_(*x*_*i*_))}, where *x*_*i*_ ∈ *U*, μ_*A*_:*U* → [0, 1] is the membership function of *A*, and μ_*A*_(*x*) ∈ [0, 1] is the degree of membership of *x* in *A*. Such fuzzy sets are called type-I fuzzy sets. However, this kind of fuzzy sets is unable to model different types of uncertainties since their membership functions are crisp. Membership functions of type-II fuzzy sets are on the other hand fuzzy and can model different types of uncertainties. A type-II fuzzy set *A*′ is characterized by a type-II membership function $\mu_{A^{\prime }}\left(x,\mu \right)$, where *x* ∈ *U* and μ ∈ [0, 1], and is defined as:

(3) $$\begin{array}{l} A^{\prime }=\left\{\left(x,\mu \right),\mu_{A^{\prime }}\left(x,\mu \right)|\forall x\in U,\mu \in \left[0,1\right]\right\} \end{array}$$

where $0\leq \mu_{A^{\prime }}\left(x,\mu \right)\leq 1$.

**Figure 1:**
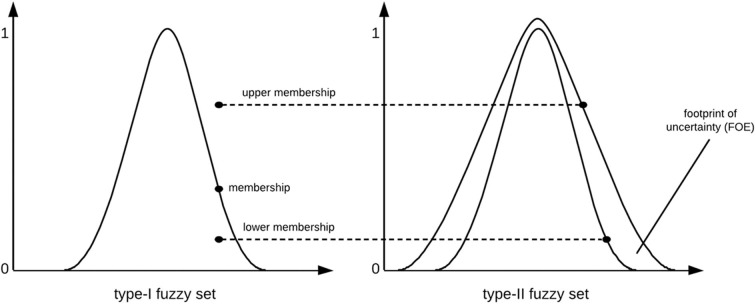
Creating a type-II fuzzy set.

Type-II fuzzy sets can be simply formed by firstly defining a type-I fuzzy set and assigning lower and upper membership degrees to each element in order to construct the *footprint of uncertainty (FOU)*, that is, the interval between the lower and upper membership values (Figure 1 depicts this concept). A type-II fuzzy set can be defined as (42):

(4) $$\begin{array}{l} A^{{}^{\prime }}=\{\left(x,\mu_{U}\left(x\right),x,\mu_{L}\left(x\right)\right)|\mu_{L}\left(x\right)\leq \mu \left(x\right)\leq \mu_{U}\left(x\right),\\ \mu \in \left[0,1\right]\} \end{array}$$

where μ_*L*_ and μ_*U*_ represent the *lower* and *upper* membership degrees of the initial membership function μ(*x*), respectively, defined as follows (42):

(5) $$\begin{array}{l} \mu_{L}\left(x\right)={\left[\mu \left(x\right)\right]}^{\alpha } \end{array}$$

(6) $$\begin{array}{l} \mu_{U}\left(x\right)={\left[\mu \left(x\right)\right]}^{\frac{1}{\alpha }} \end{array}$$

where α ∈ (1, ∞). In this paper, α = 2 since α >> 2 is not meaningful for image data (42).

## 5. Fuzzy C-Means Clustering

Let *X* = {*x*_1_, …, *x*_*i*_, …, *x*_*n*_} be the set of *n* objects (i.e., pixels), *f*(*x*_1_, *y*_1_), …, *f*(*x*_*i*_, *y*_*j*_):*i* ∈ [1, …, *m*]; *j* ∈ [1, …, *n*], and *V* = {*v*_1_, …, *v*_*i*_, …, *v*_*c*_} be the set of *c* centroids in a *p*-dimensional feature space. In fuzzy c-means (FCM), *X* is partitioned into *c* clusters by minimizing the objective function *J*:

(7) $$\begin{array}{l} J=\overset{n}{\underset{j=1}{\sum }}\overset{c}{\underset{i=1}{\sum }}{\left(u_{ij}\right)}^{m}{\left||x_{j}-v_{i}|\right|}^{2} \end{array}$$

where 1 ≤ *m* ≤ ∞ is the *fuzzifier* (set to 2 in this paper), *v*_*i*_ is the *i*^*th*^ centroid corresponding to cluster *C*_*i*_, *u*_*ij*_ ∈ [0, 1] is the fuzzy membership of *x*_*j*_ to cluster *C*_*i*_, and ||.|| is the distance norm, such that:

(8) $$\begin{array}{l} v_{i}=\frac{1}{n_{i}}\overset{n}{\underset{j=1}{\sum }}{\left(u_{ij}\right)}^{m}x_{j}\;where\;n_{i}=\overset{n}{\underset{j=1}{\sum }}{\left(u_{ij}\right)}^{m} \end{array}$$

and,

(9) $$\begin{array}{l} u_{ij}=\frac{1}{\sum_{k=1}^{c}{\left(\frac{d_{ij}}{d_{kj}}\right)}^{\frac{2}{m-1}}}\;where\;{d_{ij}}^{2}={\left||x_{j}-v_{i}|\right|}^{2} \end{array}$$

The process starts by randomly choosing *c* objects that represent the centroids (means) of the *c* clusters. Membership values *u*_*ij*_ are calculated based on the relative distance (i.e., Euclidean distance) of the object *x*_*j*_ to the centroids. The centroids *v*_*i*_ of the clusters are calculated after the memberships of all objects have been found. If the centroids at the previous iteration are identical to the centroids generated at the current iteration the process terminates (43).

## 6. Fuzzy Multilayer Perceptron (F-MLP)

**Figure 2:**
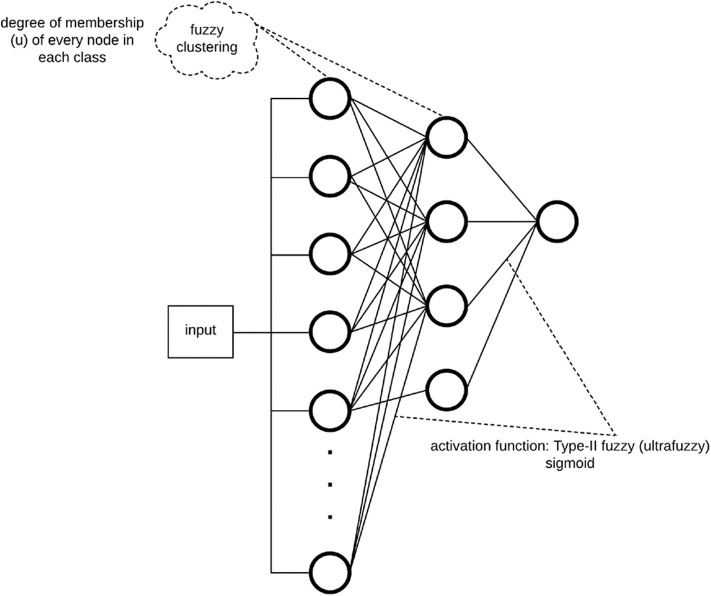
Proposed fuzzy multilayer perceptron (F-MLP) architecture.

The proposed multilayer perceptron in this paper incorporates the membership degree of each input sample to the classes of interest (e.g., regular vs. irregular) in the learning process. Moreover, the gradient descent benefits from the membership values by reducing the effects of *ambiguous* features (i.e., features that have a membership degree of 0.5) when updating the weights (learning). Membership degrees are obtained by clustering each layer in the neural network (except the output layer) using fuzzy c-means. The proposed architecture is depicted in Figure 2.

A commonly used activation function in multilayer perceptrons is the *sigmoid* activation function (Equation 10). The sigmoid function is suitable for binary classification and provides continuous values in the range [0, 1] that represent the probability of a class in the binary classification problem. As the sigmoid function introduces non-linearity in the hidden layers, it allows the neural network to learn more complex features (44).

(10) $$\begin{array}{l} sig\left(x\right)=\frac{1}{1+e^{-x}} \end{array}$$

Assuming that φ is the fuzzy sigmoid activation function, the *type-II fuzzy sigmoid* activation function can be represented as:

(11) $$\begin{array}{l} \varphi_{L}\left(x\right)={\left[\frac{1}{1+e^{-x}}\right]}^{\alpha } \end{array}$$

(12) $$\begin{array}{l} \varphi_{U}\left(x\right)={\left[\frac{1}{1+e^{-x}}\right]}^{\frac{1}{\alpha }} \end{array}$$

where φ_*L*_ and φ_*U*_ are the lower and upper sigmoid activation functions, respectively.

The proposed *fuzzy gradient descent* is defined as follows:

(13) $$\begin{array}{l} \text{w}=\text{w}-mean\left({\left|{\text{u}}_{1}-{\text{u}}_{2}\right|}^{2}\right)\times \eta \times \frac{d}{d\text{w}}\varphi \end{array}$$

where **w** are the weight values, **u**_1_ and **u**_2_ are the degrees of membership of each neuron to *class1* and *class2*, respectively; φ is the type-II fuzzy sigmoid function, and *mean* is used to represent the square differences between the degrees of membership with a single value, which can be perceived as an *ambiguity* parameter. Notice that for ambiguous nodes ${\left|{\text{u}}_{1}-{\text{u}}_{2}\right|}^{2}$ will evaluate to 0, thus having no effect on how weights are being updated. Incorporating degrees of membership in optimization will determine how input samples contribute to the learning process based on their ambiguity, such that more ambiguous features will have less effect on learning, and will rather be based on more non-ambiguous features The cost function used in our work is simply represented as the difference between the actual values and the predicted values. The F-MLP algorithm code has been open sourced and can be accessed via https://github.com/abderhasan/F-MLP.

## 7. Skin Lesion Segmentation

**Figure 3:**
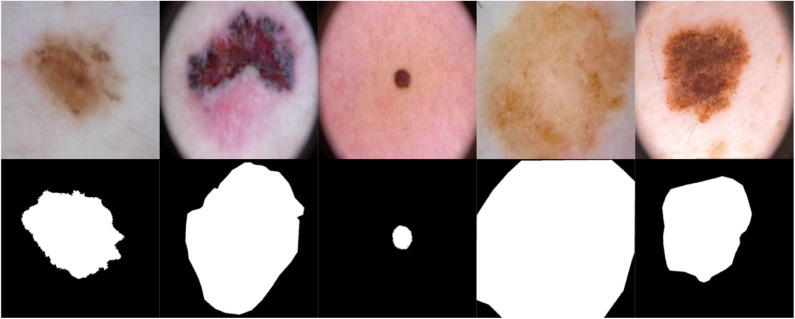
Samples of images used to train U-Net along with their groundtruth.

**Figure 4:**
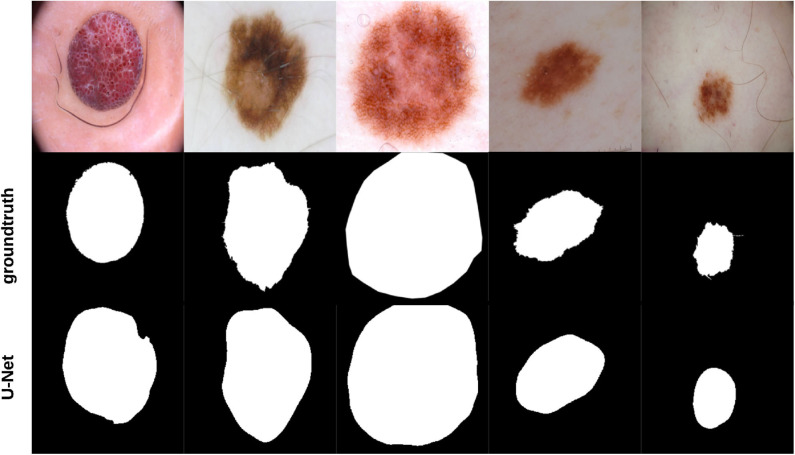
Samples of images used to test U-Net, their groundtruth, and segmentation results.

To segment skin lesions (62) we use the U-Net architecture (45, 63), an end-to-end encoder-decoder network for semantic segmentation which was firstly used for medical image segmentation. U-Net has also been used for skin lesion segmentation in dermoscopic images (46, 47). The architecture consists of two sides: left (down) and right (up). The *down* part is the encoder part [follows the Convolutional Neural Network—CNN architecture (48)] where convolution blocks are applied followed by max-pooling in order to encode the input image into feature representations at multiple levels, provided that the number of feature channels are doubled at each downsampling step. In the *up* part, the feature map is upsampled and a convolution operation is applied, bringing the number of feature channels to half; a concatenation with the corresponding cropped feature map from the *down* part occurs, followed by two 3 × 3 convolutions which are also followed by two ReLU operations and one 2 × 2 max-pooling operation with stride 2 used for downsampling. Since border pixels are lost at each convolution, the cropping process is deemed essential. The higher resolution features from the *down* part are concatenated with the upsampled features in order to localize and learn representations better. The resulting architecture is one where the expansive path is symmetric to the concatenating path, yielding a U-shaped architecture. The network is composed of 23 convolutional layers in total and does not have any fully connected layers. The final layer of U-Net uses a 1 × 1 convolution to map each 64 feature vector to the desired number of classes. An *overlap-tile strategy* is used to predict the pixels of the border region where the missing context is extrapolated by mirroring the input image. The U-Net architecture was trained for 20 epochs on a Tesla P100 GPU on 1777 dermoscopy images (resized to 256 × 256 pixels) along with their corresponding groundtruth response masks from the “ISIC 2018: Skin Lesion Analysis Toward Melanoma Detection” grand challenge datasets (49, 50), and tested on 158 images from the same dataset (those images were not used in training U-Net). Training U-Net and testing it took 27.6 min and 25.9 s, respectively. Figure 3 shows samples of the training dataset along with their groundtruth, and Figure 4 shows samples of the segmentation results using U-Net (i.e., test dataset). The average Dice similarity achieved on the 158 images was 83.8%.

## 8. Skin Lesion Border Detection

To detect the skin lesion border, we use the method we proposed in Ali et al. (46), namely *FuzzEdge*. Say that we have an image *G* of size *M* × *N* pixels with *L* gray levels: *G* = [*g*(*i, j*)]_*M* × *N*_, where *g*(*i, j*) ∈ {0, 1, …, *L* − 1} refers to a pixel in the image. Let *X* = [*x*(*i, j*)]_*M* × *N*_ be the original input image, and *Y* = [*y*(*i, j*)]_*M* × *N*_ be the filtered output image; *y*(*i, j*) = *FuzzEdge*(*X*(*i, j*)) represents the (*i, j*)^*th*^ pixel of the filtered image *Y*, where *X*(*i, j*) is a 3 × 3 kernel centered at the input pixel *x*(*i, j*) that will be affected by the filter, and *FuzzEdge*(.) is the fuzzy filter function. Three fuzzy concepts (sets) are defined: *Bright, Dark*, and *Median*. Those concepts can be created using Algorithm.1.

**Algorithm 1:** Fuzzy concept creation

	**input** : grayscale image I
	**output**: fuzzy set (concept)
**1**	For the fuzzy concepts *Bright*, *Dark*, and *Median*, specify the intervals of [*Bright*_*begin*_, *Bright*_*end*_], [*Dark*_*begin*_, *Dark*_*end*_], and [*Median*_*begin*_, *Median*_*end*_], respectively.
**2**	Let $Bright_{begin}=\left(N_{f}-1\right)\left[\frac{L-1}{N_{f}}\right]$, $Dark_{end}=\left[\frac{L-1}{N_{f}}\right]$, *Median*_*begin*_ = *Dark*_*end*_−*left*_*overlap*, and *Median*_*end*_ = *Bright*_*begin*_+*right*_overlap /* N_*f*_ *is the number of fuzzy concepts, and left*_*overlap and right*_overlap determine the overlapping range of the fuzzy concepts (the overlap range was set to 0 in this paper) */.
**3**	Set *Dark*_*begin*_ to be the first *g*_*k*_ from 0 to *Dark*_*end*_.
**4**	Set *Bright*_*end*_ to be the last *g*_*k*_ from *Bright*_*begin*_ to *L* − 1.
**5**	In interval [*Dark*_*begin*_, *Dark*_*end*_], find a pixel *g*_*k*_ with the maximum value of *p*(*g*_*k*_).
**6**	For the fuzzy concept *Dark*, create its membership function *f*_*Dark*_ as follows: *m*_*Dark*_ ← *g*_*k*_, α_*Dark*_ ← *m*_*Dark*_ − *Dark*_*begin*_, β_*Dark*_ ← *Dark*_*end*_ − *m*_*Dark*_.
**7**	In interval [*Median*_*begin*_, *Median*_*end*_], find a pixel *g*_*k*_ with the maximum value of *p*(*g*_*k*_).
**8**	For the fuzzy concept *Median*, create its membership function *f*_*Median*_ as follows: *m*_*Median*_ ← *g*_*k*_, α_*Median*_ ← *m*_*Median*_ − *Median*_*begin*_, β_*Median*_ ← *Median*_*end*_ − *m*_*Median*_.
**9**	In interval [*Bright, begin* − *Bright*_*end*_], find a pixel *g*_*k*_ with the maximum value of *p*(*g*_*k*_).
**10**	For the fuzzy concept *Bright*, create its membership function *f*_*Bright*_ as follows: *m*_*Bright*_ ← *g*_*k*_, α_*Bright*_ ← *m*_*Bright*_ − *Bright*_*begin*_, β_*Bright*_ ← *Bright*_*end*_ − *m*_*Bright*_.

**Figure 5:**
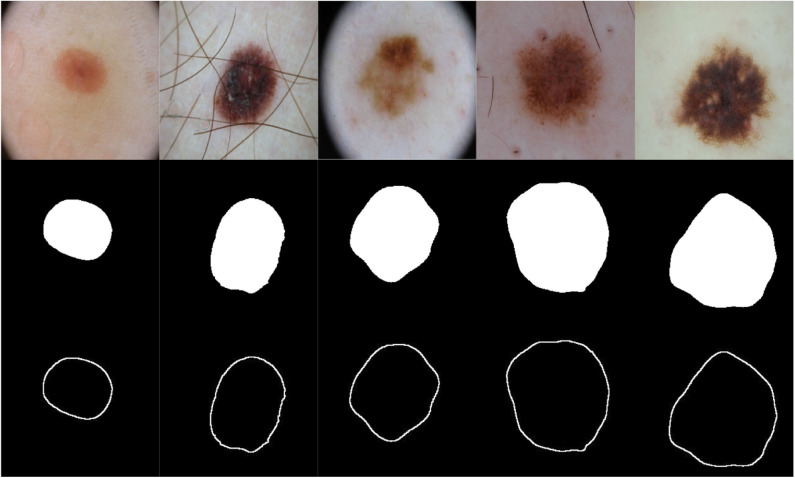
Samples of test images, their segmentations using U-Net, and borders detected using FuzzEdge.

The value associated with each pixel in the image is determined using the membership function (i.e., *f*_*Median*_) of the corresponding fuzzy concept. FuzzEdge runs three standard deviation processes to determine the values of the filtered pixel (center pixel in the kernel) on each fuzzy concept, producing three values for each pixel: $\bar{y_{Bright}}\left(i,j\right)$, $\bar{y_{Dark}}\left(i,j\right)$, and $\bar{y_{Median}}\left(i,j\right)$. In the decision step of FuzzEdge, a standard deviation process similar to the above is applied on the pixels through a kernel, provided that the pixel values will be determined using a new membership function, that is, the fuzzy interval membership function. The final output of each filtered pixel is eventually determined by measuring the distance between $\bar{y_{Bright}}\left(i,j\right)$, $\bar{y_{Dark}}\left(i,j\right)$, and $\bar{y_{Median}}\left(i,j\right)$ to the fuzzy estimator, and taking the nearest pixel to the fuzzy estimator as the value of the filtered pixel in our kernel. Figure 5 shows some samples of borders detected using FuzzEdge.

FuzzEdge code has been open sourced and can be accessed from: https://github.com/abderhasan/fuzzedge.

## 9. Skin Lesion Border Irregularity

To measure skin lesion border irregularity, a measure that combines fractal dimension and convexity [similar to that proposed in our other work (33)] is used to form an *objective* quantitative measure of border irregularity, especially that many of the signs that the clinician relies on in diagnosis involve *subjective* judgment. This applies to visual signs such as border irregularity; it has been shown that both clinicians and patients find it hard in agreeing upon whether a naevus border is considered irregular or not (51). Such measure could thus aid in improving the diagnostic accuracy.

**Figure 6:**
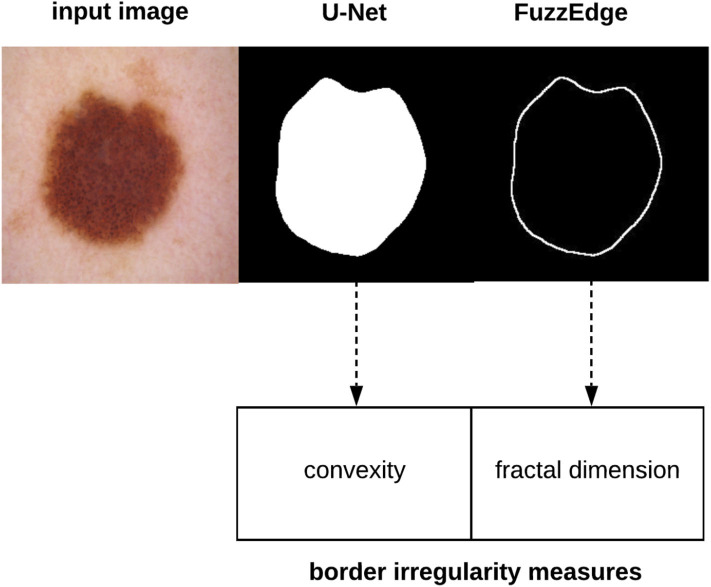
Skin lesion border irregularity measures extraction.

Figure 6 depicts the process of extracting the skin lesion irregularity measure, which also shows that our measure will be represented as a vector of two values: convexity and fractal dimension, especially that those measures have been utilized in characterizing skin lesion border irregularity in literature.

**Figure 7:**
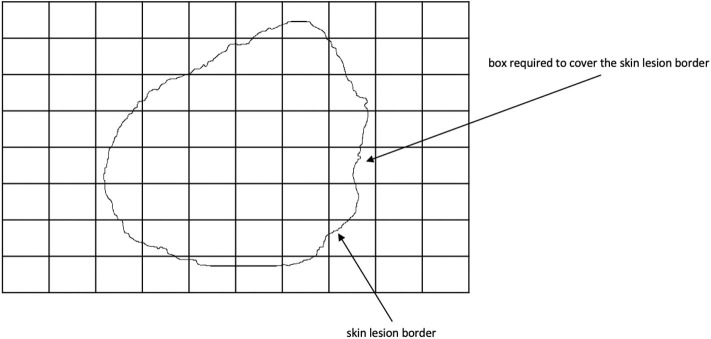
Twenty-two boxes are required to cover the skin lesion border using the box-counting method.

Fractal dimension has been used in characterizing skin lesion border irregularity as in Claridge et al. (51), Ng and Lee (30), and (52). Fractal geometry (53) describes the space-filling capacity of irregular borders which is considered size independent and does not require any smoothing operations of irregular borders for measurement to be possible (54), meaning that structures don't need to possess a perfect geometric shape. Fractal dimension is a mathematical parameter that is able to quantify the irregularity of a skin lesion border via an objective observer-independent value, such that a higher fractal dimension refers to a higher degree of complexity of the analyzed pattern. A straight line in a 2-dimensional system has a fractal dimension of *one*, and more complicated lines (having fractal properties) will have larger dimensions (55). The fractal dimension is able to describe melanoma irregular borders that possess fractal properties more accurately than Euclidean measures (i.e., perimeter) (56). The *box-counting* method (57) is used to estimate the fractal dimension of the skin lesion border, and is defined as:

(14) $$\begin{array}{l} D=\underset{e\to 0}{\lim}\frac{\log N\left(e\right)}{\log\left(\frac{1}{e}\right)} \end{array}$$

where *D* = [1, 2] is the box-counting fractal dimension of the skin lesion border, *e* > 0 is the side (edge) length of the box, and *N* is the smallest number of boxes of side length *e* needed to completely cover the skin lesion border. The fractal dimension is the *slope* in the $\log N\left(e\right)/\log\left(\frac{1}{e}\right)$ graph. Figure 7 demonstrates the box-counting method.

The straighter the skin lesion border the lower the value *D*, and vice versa. As melanoma borders tend to be irregular they are considered to be similar to fractals [i.e., Koch snowflake (58)] and are expected to have a higher fractal dimension than regular-boundary naevi. It was found in Cross et al. (54) that the fractal dimension of all lesions are greater than the topological dimension (i.e., one), indicating the existence of a fractal element in their structure.

Convexity, the ratio between the perimeter (the number of points/length of the boundary) of the convex hull of the skin lesion (the smallest convex polygon that surrounds all of the skin lesion pixels) and the skin lesion perimeter, can be used to characterize the skin lesion border shape and irregularity (37, 59, 60). Convex objects tend to have a convexity value of 1, as opposed to non-convex objects (i.e., irregular skin lesion borders) which tend to be less than 1. In other words, convexity shows the amount by which the object differs from the convex object.

## 10. Results and Discussion

**Figure 8:**
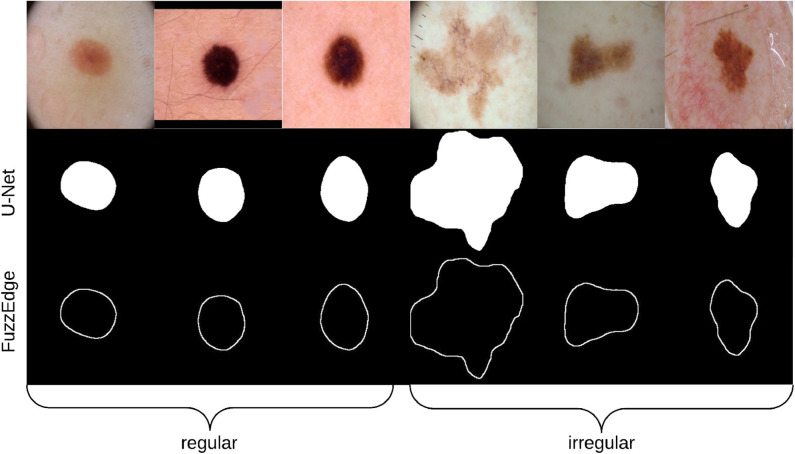
Samples of regular and irregular borders labeled by the dermatologist.

To prepare the training and testing data for F-MLP, 158 images segmented using U-Net were used, their skin lesion borders extracted using FuzzEdge, and the extracted borders sent to a dermatologist (Dr.Sally O'Shea) to label as *regular* or *irregular* borders (regular: 5, irregular: 153), which will eventually serve as our groundtruth (labels) for the training data. Figure 8 shows some samples of regular and irregular borders along with their original and segmented images. However, due to the imbalance in data, an *augmentation* step (rotating, and flipping horizontally and vertically) has been carried out to increase the regular samples. Augmentation was carried out on the 5 regular bordered images, producing multiple versions of those images. The total number of images after augmentation was 310 images (regular: 157, irregular: 153).

**Figure 9:**
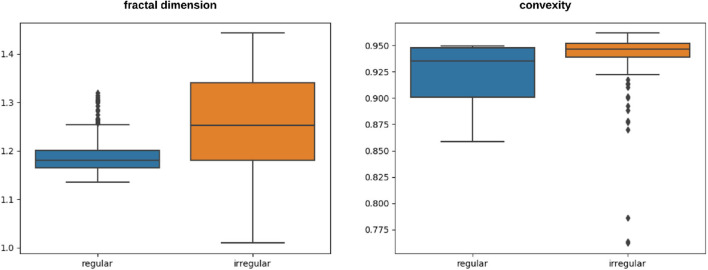
Box-and-whisker plots representing the fractal dimension and convexity distributions of the skin lesions (regular and irregular) used in training and testing the neural network.

Table 1 shows the fractal dimension and convexity values for the images in Figure 8. It should be emphasized that fractal dimension is found for the edge images (i.e., FuzzEdge) and the convexity is found for the segmentation (i.e., U-Net) results of the image, as demonstrated in Figure 6. Figure 9 shows box-and-whisker plots depicting the distribution of fractal dimension and convexity values for the regular and irregular skin lesions used in training and testing the neural networks.

**Table 1:** Border irregularity measures for the images presented in Figure 8.

**Image**	**Fractal dimension**	**Convexity**	**Label**
1.r	1.2527	0.9898	1
2.r	1.2599	0.9890	1
3.r	1.2875	0.9893	1
1.i	1.4499	0.9031	0
2.i	1.3056	0.9531	0
3.i	1.3125	0.9586	0

*Images 1.r, 2.r, and 3.r from left to right refer to the first three images (regular), and images 1.i, 2.i, and 3.i refer to the last three images (irregular)*.

The extracted skin lesion border irregularity measures were used to train and test a standard neural network and a type-II F-MLP. For both networks the number of neurons in the input layer is equal to the number of input features (2 features), the first hidden layer is composed of 4 neurons, the second hidden layer is composed of 2 neurons, the output layer is composed of 1 neuron which represents the final classification result, and the learning rate is 0.001. Experiments were run on a machine with an Intel Core i7 processor of speed 2.2 GHz and 16 GB memory.

After obtaining the *prediction probability* ∈ [0, 1] of each test sample, a *threshold* is generated from the prediction probabilities to decide the final prediction (regular or irregular) according to Equation (15).

(15) $$\begin{array}{l} threshold\;=\frac{\sum_{i=1}^{n}p_{i}}{n} \end{array}$$

where *n* is the number of test samples, and *p*_*i*_ is the prediction probability of test sample *i*. The final decision is obtained using Equation (16).

(16) $$\begin{array}{l} Decision=\{\begin{array}{cc} regular, & \;p_{i}>threshold\;\\ irregular, & \;p_{i}\leq threshold\; \end{array} \end{array}$$

Tables 2, 3 show the different training and testing split ratios used to evaluate the networks, number of iterations used in each network, time consumed (in seconds), and accuracy, for the standard neural network and F-MLP, respectively. The networks were run for only 1 iteration since more iterations didn't improve the accuracy.

**Table 2:** Standard neural network evaluation on classifying regular and irregular borders using different training and testing split ratios.

**Ratio**	**Training**	**Testing**	**Training time**	**Testing time**	**Accuracy (%)**
80:20	248	62	0.02	0.007	91.9
70:30	217	93	0.02	0.008	91.4
60:40	186	124	0.01	0.007	87.9
50:50	155	155	0.02	0.009	79.4

**Table 3:** F-MLP evaluation on classifying regular and irregular borders using different training and testing split ratios.

**Ratio**	**Training**	**Testing**	**Training time**	**Testing time**	**Lower sigmoid acc. (%)**	**Upper sigmoid acc. (%)**
80:20	248	62	0.58	0.01	95.2	90.3
70:30	217	93	0.8	0.07	91.4	89.2
60:40	186	124	0.6	0.08	90.3	87.9
50:50	155	155	0.7	0.08	83.9	75.5

Using two sigmoid activation functions reflects the type-II fuzzy set in that the error rates represent the range of performance that could be achieved using the fuzzy neural network (F-MLP), modeling thereby the potential uncertainty occurring within the input data. Two versions (lower and upper sigmoid) of F-MLP can be obtained, and the one with the best performance (maximum accuracy) can be used as shown in the following equation:

(17) $$\begin{array}{l} \lambda FMLP=\max\left(\lambda FMLP_{lower},\lambda FMLP_{upper}\right) \end{array}$$

where λ*FMLP* is the accuracy of the fuzzy multilayer perceptron, λ*FMLP*_*lower*_ is the accuracy of the fuzzy multilayer perceptron utilizing the lower sigmoid activation function, and λ*FMLP*_*upper*_ represents the accuracy of the fuzzy multilayer perceptron utilizing the upper sigmoid activation function.

**Figure 10:**
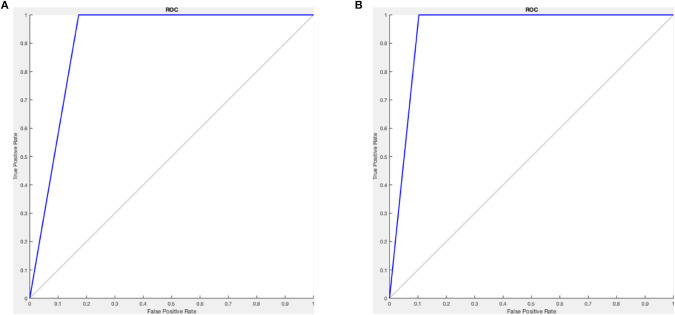
**(A)** Standard neural network ROC curve **(B)** F-MLP (lower sigmoid) ROC curve.

In comparing the standard neural network and F-MLP, we consider the 80:20 ratio as it results in better accuracy amongst the other ratios, evaluating to 91.9 and 95.2% for the standard neural network and F-MLP, respectively. Tables 4, 5 depict the confusion matrices of the classification results, from which we derive the sensitivity and specificity values that evaluate to 100 and 82.8% for the standard neural network, respectively, and 100 and 89.7% for the F-MLP, respectively. Figure 10 depicts the receiver operating characteristic (ROC) curves of the standard neural network and F-MLP.

**Table 4:** Standard neural network confusion matrix.

		**Predicted**		
		**Regular**	**Irregular**	**Total**
Actual	Regular	33	0	33
	Irregular	5	24	29
	Total	38	24	62

**Table 5:** F-MLP (lower sigmoid) confusion matrix.

		**Predicted**		
		**Regular**	**Irregular**	**Total**
Actual	Regular	33	0	33
	Irregular	3	26	29
	Total	36	26	62

To evaluate the proposed approach further, we compare it with other state-of-the-art classification methods as shown in Table 6 which shows that F-MLP outperforms most of those methods.

**Table 6:** F-MLP evaluation on classifying regular and irregular borders using different training and testing split ratios.

**Method**	**TP**	**TN**	**FP**	**FN**	**Accuracy (%)**
F-MLP (lower sigmoid)	33	26	3	0	95.2
Stochastic gradient descent	33	26	3	0	95.2
Random forests	32	28	1	1	96.8
Logistic regression	32	22	7	1	87.1
K-nearest neighbors	32	26	3	1	93.5
Gaussian naive bayes	32	22	7	1	87.1
Support vector machine	28	23	11	0	87.1
Decision tree	32	26	3	1	93.5

Incorporating the membership degree in the gradient descent (Equation 13) helps in reducing the effects of ambiguous features/neurons when updating the weights, and thus increases the performance of learning (i.e., higher accuracy predictions). The proposed type-II F-MLP is able to perform better than its traditional neural network counterpart with fewer iterations. However, training F-MLP is more time-consuming than its traditional neural network counterpart.

## 11. Conclusion

An automatic approach for detecting the skin lesion border irregularity has been proposed. The approach starts by segmenting the skin lesion using U-Net, detecting the lesion border using FuzzEdge, extracting the irregularity measures (fractal dimension and convexity), training a F-MLP on the extracted measures, and predicting border irregularity on new images using the trained model. The proposed F-MLP utilizes type-II fuzzy sets and showed to provide better prediction accuracy than most of the state-of-the-art classification methods in general and its standard neural network counterpart in particular.

The proposed approach reflects three main contributions: (i) developing a fuzzy gradient descent that considers the membership degrees of neurons, minimizing thereby the effects of ambiguous neurons on the neural network learning process, (ii) proposing a type-II fuzzy sigmoid activation function which allows to represent the range (lowest and highest) of performance the fuzzy neural network is able to attain, where the fuzzy neural network with the highest performance (highest accuracy) could be utilized in the prediction process, (iii) proposing an irregularity measure that is represented as a vector of fractal dimension and convexity values. The approach is however more time-consuming when training the neural network.

The process of assigning regular and irregular labels to the skin lesion borders in the F-MLP training phase is considered laborious and might involve a larger team to be able to label thousands of lesion borders, a task that could eventually improve the prediction accuracy.

This work leads us to what we call fuzzy deep learning, in which we hypothesize that it would improve the traditional deep learning approaches currently used in literature. We are aiming to investigate further the combination of fuzzy logic and CNNs. There could be different approaches to fuzzifying the CNN such as using fuzzy arithmetic instead of the currently used convolution arithmetic (61) for instance. This will be demonstrated further in a future study.

As future work, we would also like to investigate introducing more metrics in the skin lesion border irregularity measure vector to increase the robustness of such measure. Moreover, we would like to apply the method on problems incorporating more than two classes, and on skin lesion images taken using a mobile phone camera (i.e., less quality) as opposed to dermoscopic images.

## Data Availability Statement

All datasets generated for this study are included in the article/supplementary material.

## Author Contributions

A-RA was the main author of the paper, designed and implemented the method, and drafted the paper. JL was the main author's Ph.D. supervisor and provided deep analytical thoughts, suggestions, comments, and feedback that improved the paper dramatically. GY and SK provided comments and feedback on the paper and raised some questions about the proposed method. AH provided comments and feedback on the paper and raised some questions about the proposed method and how to improve it. SO'S acted as the dermatologist who annotated our skin lesion border images which were used as a ground truth throughout our experiments. All authors contributed to the article and approved the submitted version.

## Conflict of Interest

The authors declare that the research was conducted in the absence of any commercial or financial relationships that could be construed as a potential conflict of interest.
